# Sperm activation through orbital and self-axis revolutions using an artificial cilia embedded serpentine microfluidic platform

**DOI:** 10.1038/s41598-018-22563-8

**Published:** 2018-03-15

**Authors:** Bivas Panigrahi, Chang-Hung Lu, Neha Ghayal, Chia-Yuan Chen

**Affiliations:** Department of Mechanical Engineering, National Cheng Kung University, Tainan, 701 Taiwan

## Abstract

The zebrafish sperm activation profoundly depends upon the homogeneous mixing of the sperm cells with its diluent in a quick succession as it alters the cell’s extracellular medium and initiates their motility. Manual stirring, the traditional method for zebrafish sperm activation is tedious, time-consuming, and has a poor outcome. In this aspect, an artificial cilia embedded serpentine microfluidic is designed through which hydrodynamic factors of the microfluidic environment can be precisely regulated to harness uniform mixing, hence ensuring a superior sperm activation. To quantify the sperm motility, computer assisted sperm analysis software (CASA) was used whereas to quantify the generated flow field, micro particle image velocimetry (μPIV) was used. With this proposed microfluidic, 74.4% of the zebrafish sperm were activated which is 20% higher than its currently existing manual measurements. The μPIV analysis demonstrates that the curvature of the microchannel induces an orbital rotation to the flow field along the length of the microchannel together with the artificial cilia actuation which instigates a local rotation to the flow field of the artificial cilia location. The collective rotation in the whole flow field induce vorticity that promotes the change in temporal dynamics of the sperm cells towards their activation.

## Introduction

Zebrafish is considered as an invaluable animal model because of its experimental friendly attributes. It has been extensively used in the current laboratory environment to understand the vertebrate development^1,2^, drug discovery^3^, genetic research^4^, and disease modelling^5^. To meet experiment demand, zebrafish sperms are collected in the zebrafish core facilities around the world and used for *In vitro* reproduction^6^. Zebrafish is a fresh water fish whose sperm get activated quickly under low osmotic conditions^7^. In regards to sperm activation, transmembrane cell signalling plays a crucial role as the low osmotic condition which promotes the Ca^2+^ influx through the respective channel via the plasma membrane of the sperm cells and increases the intracellular Ca^2+^ concentration that further promotes sperm motility^8^. During normal mating, zebrafishes are right next to each other, and there is no issue with this short time window of motility. However, during *in vitro* reproduction, even a small time lag may reduce reproductive success. In the current lab environment for *in-vitro* reproduction, zebrafish sperms are manually activated through hand mixing method which have a poor outcome.

A microfluidic cell manipulation technique opens a new window to the field of bioengineering as it significantly reduces the sample volume, processing time, and moreover, offers high throughput capacity and increased reproducibility^3^. In the recent years, microfluidic devices have been employed to study the sperm behavior^9,10^. When it comes to understand the zebrafish sperm activation, in the recent years, few microfluidic devices in conjunction with CASA (Computer-assisted sperm analysis) have been employed to activate and quantify the motility of zebrafish sperm cells^11–13^. Apparently, in an environment of low Re-number, where viscous force dominates over the inertial force, it has always been a challenge to attune superior mixing in a shorter microchannel length. In this aspect, both passive^11,12^ and active^13^ micromixers have been employed towards sperm cell activation. Park *et al*. 2012 had proposed a staggered herringbone type micromixer for zebrafish sperm cell activation which can be considered as the first footprint towards microfluidic based zebrafish sperm cell activation. In the following years, Scherr *et al*. 2015 proposed a SeLMA (sequential logarithmic mixing apparatus) through which extracellular medium of sperm cells can be rapidly diluted. As a result, motility of the sperm cells is initialized^11^. They coupled the experiment with the numerical simulation to reach to an opinion that intracellular ion concentration needs to be adjusted for standardized high throughput zebrafish sperm cell activation. Recently, our group had proposed an active type micromixer for the superior activation of zebrafish sperm cells^13^. To further improve the sperm activation rate a new hydrodynamic concept is introduced and discussed in this study which is described in the following.

Several hydrodynamic factors such as fluid shear^14,15^, vorticity^16^ etc. substantially influence the sperm characteristics and morphologies and promote individual sperm activation. For an efficient and high throughput zebrafish sperm activation, the microfluidic device needs to be designed in such a way that the hydrodynamic factors on the sperm cells can be precisely controlled. Moreover, considering the buoyant nature and short life span of the zebrafish sperm, uniform yet in-plane mixing profile is desired within the microfluidics in a very short time period^17^. To address these aforementioned matters of interest, an artificial cilia embedded serpentine microfluidic device was designed in this work. It was hypothesized that the curvilinear design of the serpentine microfluidic device will generate a global three dimensional velocity field and subsequently, induce an orbital rotation to the whole flow field. Combing this generated global rotation of the flow field with local fluid rotation induced through the self-axis revolution of artificial cilia, a superior mixing can be achieved within the microfluidic device. This enhanced micromixing phenomenon will expeditiously alter the temporal dynamics of the sperm cells and initiate their motility.

## Material and Methods

### Zebrafish sperm collection

To maintain the genetic similarity during the experiment, the zebrafish sperms were collected from a batch of single transgenic zebrafish line Tg (gata1: dsRed) which were nurtured in our lab through a well-regulated aquatic environment (AZ-303; GENDANIO, Taiwan). To collect the zebrafish sperm for experimental use, an established protocol was followed and the details can be found elsewhere^18,19^. Hank’s balanced salt solution (HBSS) at an osmolality of 300 mosmol/kg was used as extender. The collected sperms were then suspended in a centrifuge tube with 100 μL (∼10–20 times of the sperm volume) of HBSS solution. It can be noted that the sperm cells were diluted in this aforementioned manner to maintain the sperm concentration of ∼1 × 10^7^ cells/mL for reliable CASA measurements^12,20^. The centrifuge tubes were then stored at a temperature of 4 °C within an insulated container. For experimental purpose, 3 μl, sperm solution were extracted through a micropipette and DI water of same volume were added to reduce the osmolality to ∼150 mosmol/kg for sperm activation^11,12^.

### Microfluidic Design and Fabrication

**Figure 1:**
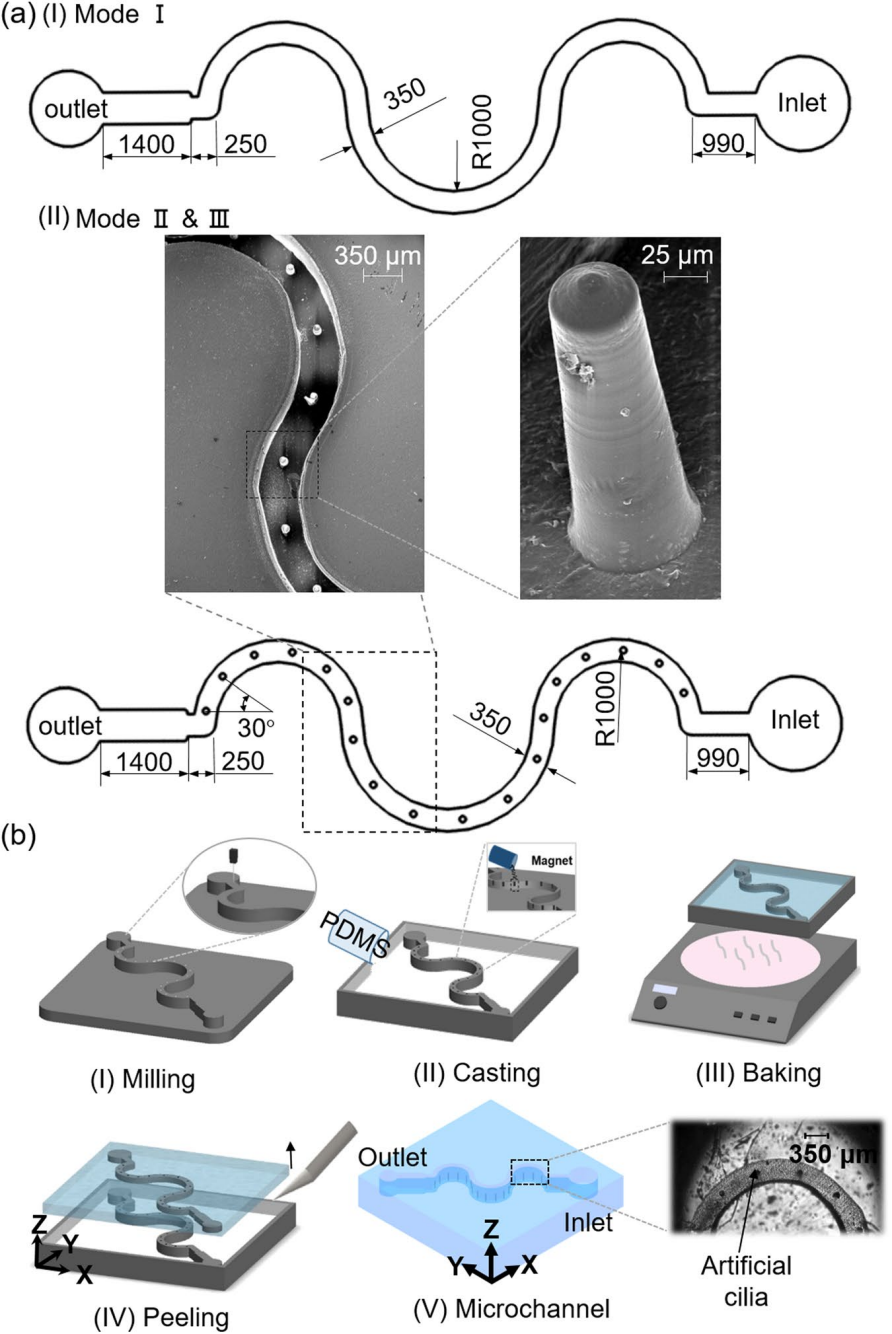
(**a**) Schematic illustration as well as the dimensional details of the serpentine microchannel. Three modes describe the three different experimental conditions. For Mode I: microchannel without cilia, Mode II: microchannel with static cilia, and for Mode III: microchannel with cilia rotating in a clockwise manner. All the dimensions are in μm and the figures are not up to scale. The inset picture depicts the SEM picture of the section of a microchannel and the artificial cilia structure. (**b**) Schematic depiction of the microfabrication procedure.

Two types of the serpentine microfluidic device, namely, with and without artificial cilia, were used for this study. To demonstrate the improvement of zebrafish sperm activation corresponding to structured stirring, this study was designed in three modes, namely, Mode I (microchannel without cilia), Mode II (microchannel with static cilia), and Mode III (microchannel cilia rotating in a clockwise manner). It can be noted that for all the three modes, the design and dimension of the microfluidic channel were kept constant (Fig. 1a). The stepwise fabrication process flow layout is illustrated in the Fig. 1b. To prepare the mold of the microchannel, an acrylic sheet was used, and the design of the microchannel was engraved on to it through a series of computerized numerical control (CNC) machining. For the mold of artificial cilia, holes of 400 μm height and diameter of 50 μm were drilled on to the acrylic’s surface. To prepare for the structure of the artificial cilia, a homogeneous mixture of neodymium-iron-boron particles (MQP-15-7, Magnequench, Singapore) with the polydimethylsiloxane (PDMS, sylgard 184: Dow Corning corp., Midland, USA) in a ratio of 1:4 was prepared, and introduced into the drilled holes. Once the holes were completely filled, a homogeneous mixture of the PDMS and curing agent in a proportion of 10:1 were introduced into the mold to prepare the base and wall of the microchannel. The mixture was degassed prior to its introduction into the mold so that any entrapment of air can be removed. Subsequently, the complete structure consisting PDMS and mold were subjected to the hot plate baking at a temperature of 95 °C for approximately 4 hours to obtain the elasticity of the PDMS channel. Then, cured PDMS channel was removed from the parent mold and the experiment was carried out.

### Magnetic actuation system

**Figure 2:**
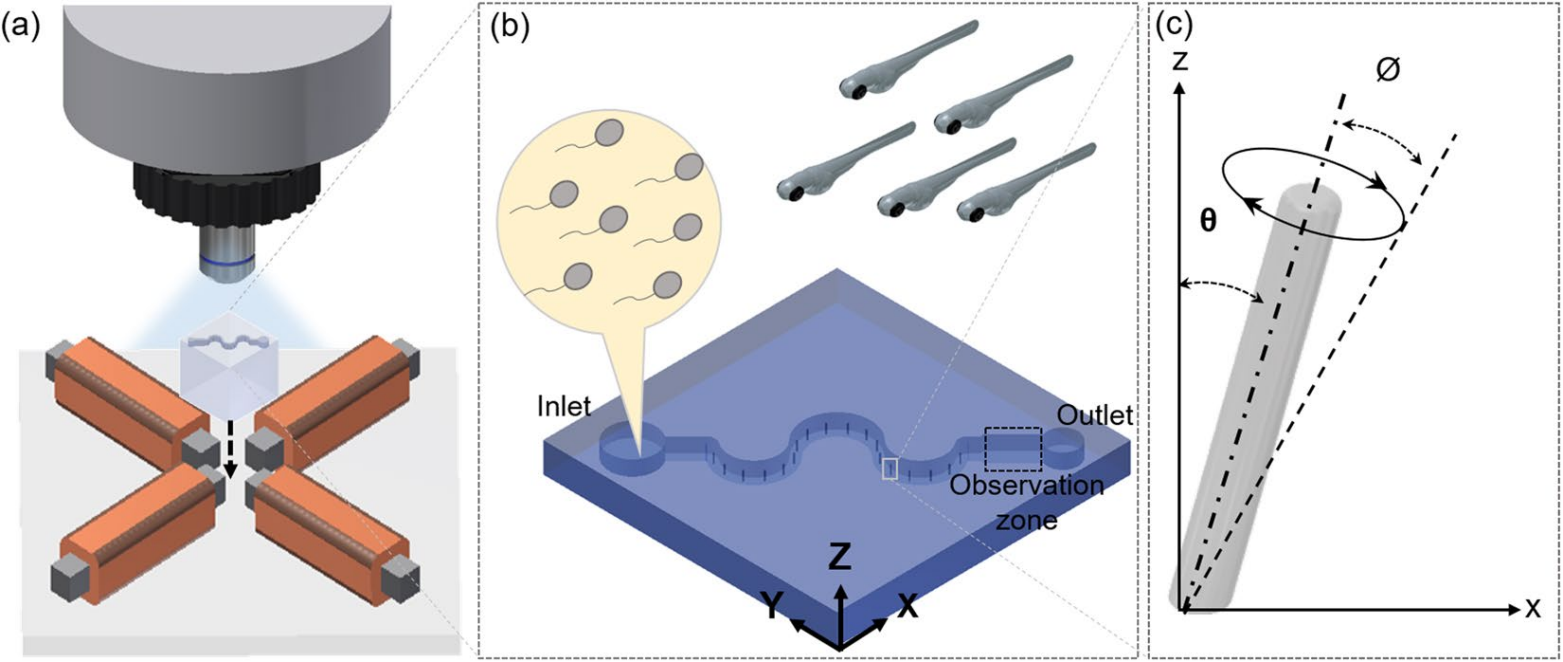
(**a**) A magnetic actuation system which consists of four electromagnetic coils to control motions of artificial cilia inside a microfluidic platform. (**b**) Schematic illustration of the microchannel, where the observation zone was in the vicinity of the outlet and reserved for the sperm motility analysis. (**c**) The artificial cilia were tilted up to a certain angle due to residual stress generated during the microfabrication procedure. All the artificial cilia were actuated in a circular manner.

For this experiment, the artificial cilia were designed to be actuated in a circular manner which have been observed naturally in the embryonic primary cilia^21^, and mimicked in a microfluidic environment to generate flows as well as well as mixing operation^22,23^. To generate such trajectory, a homogeneous magnetic field is indeed necessary. Therefore, an experimental setup was designed that is comprised of four electromagnetic coils mounted on to a horizontal plane, external power supply (GPR-3510HD DC power supply, Instek, Taiwan), data acquisition system (NI cDAQ-9174, National Instruments, Austin, TX, USA), and a customized graphic user interface (Fig. 2). For the electromagnetic coils, a 24-gauge copper wire was wrapped around a rectangular iron bar and both ends of the wires were connected to the power supply through the data acquisition system with embedded modules for both signal inputs and outputs. The current was supplied to the electromagnets in a format of the pulse width modulation (PWM) wave form. The electromagnets can generate up to 0.2 T which is substantial towards the uniform actuation of the artificial cilia array. A detailed description regarding the experimental setup can be found elsewhere^23^.

### Sperm motility analysis

To perform sperm activation experiments, sperm samples were loaded into the microfluidic platforms through a micro pipette. Once the flow becomes steady, the artificial cilia were actuated in a continuous manner and the sperm activation was quantified in the observation zone which is in the vicinity of the outlet. To observe the sperm movement, a high-speed camera mounted on the microscope (BX60, Olympus Corp., Japan) with 20-X magnification lenses was employed. For a meaning full direct comparison, the same experimental condition was imposed during all the three experimental modes. To evaluate the sperm motility, an open source computer assisted sperm analysis plug-in of the Image J software was employed^24^. This software evaluates the sperm motility by quantifying the movement of sperm with respect to a steady flow environment. The images were acquired at a frequency of 30 Hz meanwhile setting the size of the sperm cell in a range of 8–12 pixels. This is a well-established methodology that was previously adopted and has been validated to quantify the sperm motility and well documented in the literature^13,24^.

### µPIV analysis

To quantify the hydrodynamics in the proposed artificial cilia embedded microchannel, micro particle image velocimetry (µPIV) analysis was carried out. Fluorescent polystyrene particles (Microgenics, Inc., Fremont, CA, USA) of 3.2 µm diameter were seeded into a glycerol aqueous solution (with 80% by weight) and introduced into the microchannel through a syringe pump. Once the flow was steady, the artificial cilia were actuated and time dependent particle motions were imaged under a fluorescent microscope (BX60, Olympus Corp., Japan). The recorded time dependent particle motions were quantified subsequently through a commercially available software (Dynamic Studio, Dantec Dynamics, Denmark). Velocity vectors were further quantified using an adaptive interrogation of 16 × 16-pixel interrogation window. Subsequently, other hydrodynamic parameters such as vorticity and circulation were quantified from the velocity vectors.

### Statistical analysis

To determine the statistical significance of different experimental modes, one way ANOVA test was performed through the commercially available MEDCALC software (MedCalc®, Ostend, Belgium). For analysis, post-hoc test with the logarithmic transformation of ‘Turkey-Kramer’ was considered and a *p*-value less than 0.05 were considered as statistically significant between the data sets.

## Results and Discussion

### Zebrafish sperm activations

**Figure 3:**
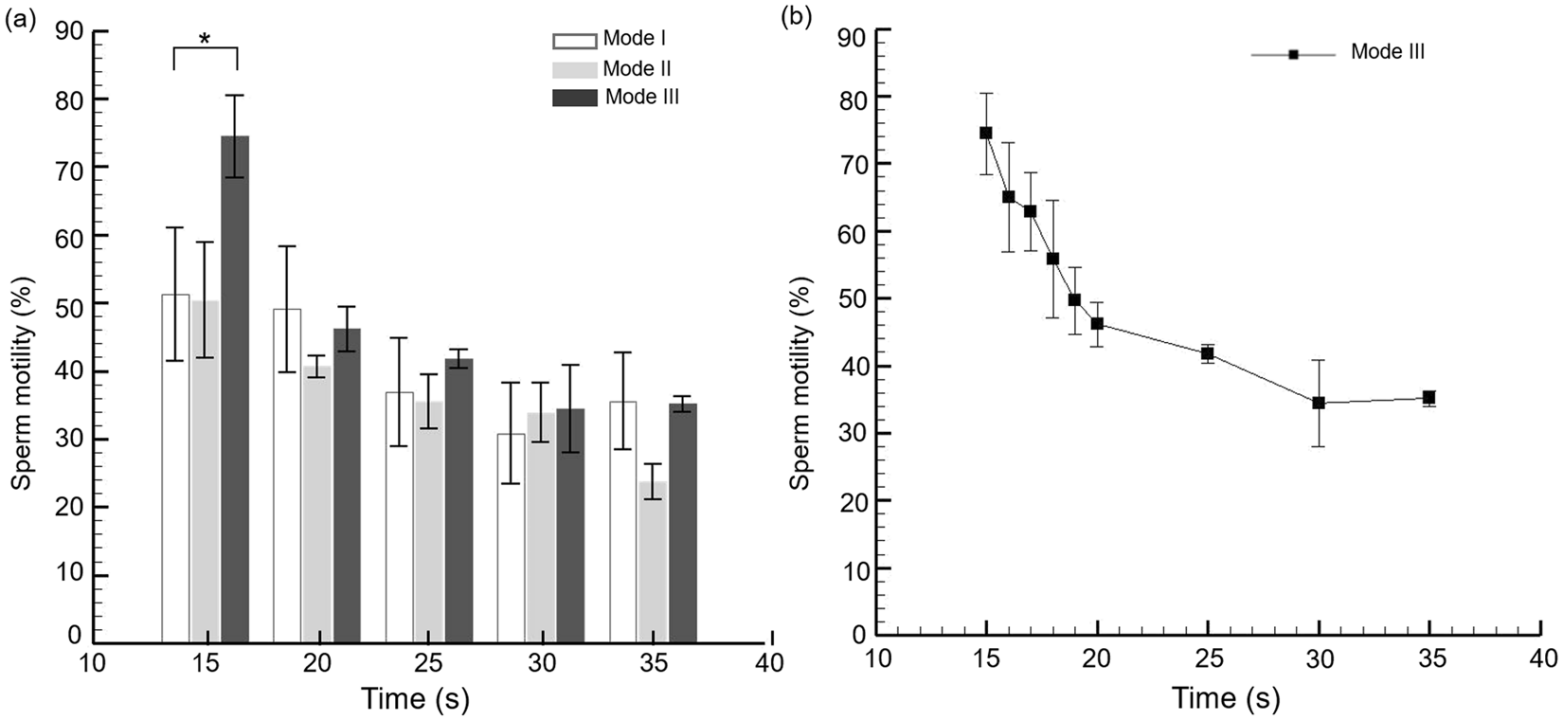
(**a**) Zebrafish sperm activation corresponding to the increase in time for the three different experimental modes. A one way ANOVA test was performed and a *p*-value < 0.05 considered to be statistically significant (*). (**b**) Time dependency of the sperm activation rate in Mode III illustrates a progressive decline in a time window of 15–20 s illustrating the importance of artificial cilia to the sperm activation.

The zebrafish sperm activation through three different experimental modes, namely, Mode I, Mode II, and Mode III, were quantified and compared corresponding to the increase in time (Fig. 3a). The sperm activation for three individual modes was recorded during the first 15 to 35 s as the first 15 s were reserved for sperm to flow from the inlet, and through the artificial cilia zone (Mode II, and Mode III) before reaching the measurement zone. Also, achieving a steady flow state within the microchannel with the purpose of initializing both the high-speed camera and computer assisted sperm analysis system (CASA) were allocated in this time window. As observed, for all the three modes, the highest sperm activation was recorded at the initial time point of 15 s. A progressive decrease after 15 s in the sperm motility was further evidenced in all of the three cases. This decrease in the sperm activation is due to the short life span of the zebrafish sperm and the downward trend of the sperm motility with respect to time is in accord with the previously reported work^11–13^. As observed, an average sperm activation for Mode III was recorded to be as high as 74.44 ± 6.07% at the initial time point of 15 s which demonstrates at least 25% increment in sperm activation compared to that in Mode I and Mode II where the average sperm activation accounted to 55.40 ± 6.39% and 50.37 ± 8.46%, respectively. On the top of that, in one test a maximum 80.75% of sperm were activated in Mode III which is much higher than its currently existing counter parts^11,13^. To test the statistical significance between three experimental modes, a one way ANOVA test was performed. A statistical significance (*p* = 0.018) was obtained between all the three modes at the initial time point of 15 s. This illustrates the importance of structured stirring towards sperm activation in a short time span, a quintessential factor towards zebrafish sperm handling. However, corresponding to the increase in time, no statistical significance was noticed among three respective modes. Moreover, for Mode III, a significant drop in the sperm activation is noticed in a time window of 15–20 s which is analyzed in details in Fig. 3b and described in the following.

In terms of time dependency of the sperm activation rate in Mode III, the progressive drop in the sperm activation is more pronounced in a time window of 15 s to 20 s when the artificial cilia were deactivated, and the average sperm activation drops to a value of 46.12 ± 3.23% from the value of 74.44 ± 6.07% (Fig. 3b). However, this progressive decline in the sperm activation exhibits a linear (*R*^2^ = *0*.*9*) relationship corresponding to the increase in time further highlighting the significance of role of activated artificial cilia to the sperm activation. As the artificial cilia were only activated from the time window of 0 s to 5 s, this significant drop in terms of sperm activation especially for the time windows over 15 s indicates that without the presence of artificial cilia actuation, the sperm activation rate dropped rapidly. It shows that the device effectively induces micromixing which is favourable towards high efficiency sperm activation. Both of these aforementioned factors including the microchannel design as well as activation of artificial cilia collectively eliminate other possible factors such as sperm cell damage due to non-uniform shear or mixing as the time discrepancy highlighted throughout. However, to this point, the underlying phenomenon which contributes to significant sperm activation in the Mode III is largely unknown and it can be hypothesized that the change in hydrodynamics due to the artificial cilia beating might be playing a crucial role in this case. To elucidate this assumption further, a comprehensive hydrodynamic analysis of the flow fields during all the three modes was further carried out as illustrated in the next section.

### Flow field hydrodynamic analysis

Apparently, in a microscopic environment of low Reynold’s (Re) number flow, viscous force dominates the inertial force making it difficult to obtain mixing through the process of diffusion. However, with the employment of artificial cilia based micromixer, a superior mixing can be achieved within a very short length scale as the artificial cilia motion induce microscale vortices that promote diffusion along the two streams of fluid^23,25^. As the intensity of the vorticity strongly relates to the micromixing operation and the sperm activation greatly depends on the micromixing^11,12^, the success of the sperm activation can be highly related to the intensity of vorticity distribution within the microchannel, as the original design of this study.

**Figure 4:**
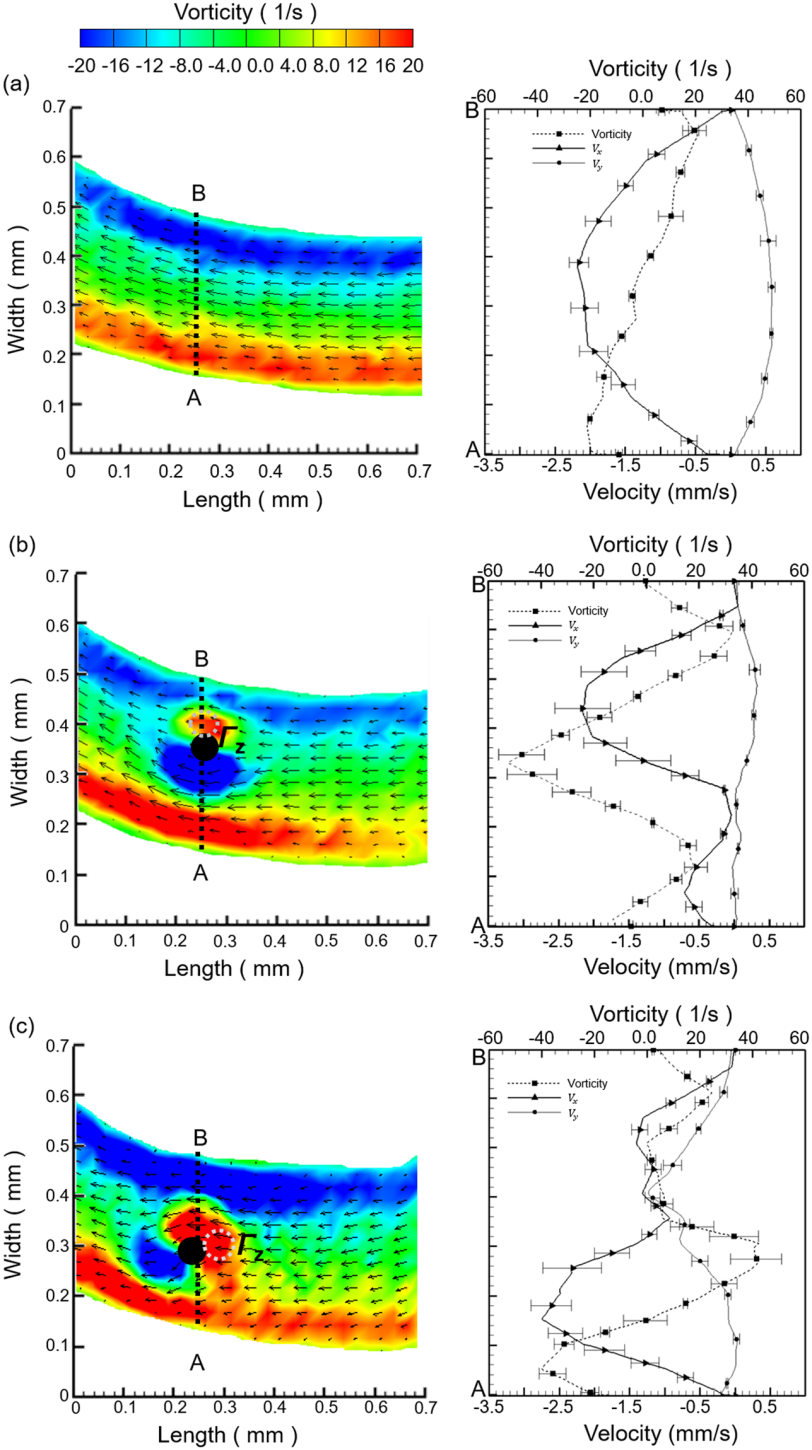
Flow patterns for (**a**) Mode I (**b**) Mode II and (**c**) Mode III in the microchannel. Left column illustrates the time ensemble vorticity contours superimposed with the ensemble average velocity. Right column illustrates the quantified vorticity and velocity along the AB line. Black circle represents the artificial cilia.

The vorticity distribution along z-direction for all the three respective modes was quantified through the horizontal plane along the artificial cilia tip by using the equation, ${\omega }_{z}=(\frac{\partial {V}_{y}}{\partial x}-\frac{\partial {V}_{x}}{\partial y})$, and illustrated in the left panel of Fig. 4. For all the three respective modes, two clearly distinguishable vortex regions of peak intensity with opposite signs were evidenced in the vicinity of the microchannel wall. This flow phenomenon can be elucidated by relating the generated velocity profile and the vorticity equation. For all the three respective modes, the change of *V*_*y*_ along x-direction $({\rm{i}}.{\rm{e}}.\frac{\partial {V}_{y}}{\partial x})$ can be neglected from the vorticity equation $({\omega }_{z})$, as the magnitude of *V*_*y*_ found to be negligible corresponding to *V*_*x*_ based on the observation of features of the laminar flow generated in the current flow conditions. For the lower half of the microchannel, the magnitude of *V*_*x*_ decreases along y-direction away from the wall (as the main flow direction is from the right hand side to the left hand side) resulting in a negative velocity gradient. Therefore it yields a net vorticity with positive magnitude. Contrast to it, a vorticity of opposite sign in the upper half of the microchannel was evidenced as the magnitude of *V*_*x*_ decreases along y direction from the microchannel center towards to the upper wall resulting in a positive velocity gradient. These vorticity distributions with opposite magnitudes near the microchannel wall apply to all three modes. However, For Mode I, the vorticity diminishes towards the center of the microchannel indicating the absence of rotation in the center of the flow field highlighting the requirement of further additive agitation. For Mode II and Mode III, along with the vorticity near the microchannel wall, two additional vortices of with opposite signs were noticed nearby the artificial cilia array. These two additional vorticity regions were induced by the parabolic velocity profile generated between artificial cilia within the microchannel. To describe the signs of these additional vertical regions, the vorticity contour data in Mode II data as shown in Fig. 4b are illustrated. There is an additional red region just below the blue region from the upper wall of the microchannel. The vorticity distribution with positive values (color in red) is caused by the nature of the velocity profile (between the upper wall and the physical presence of the cilium, and in this region the stream wised flow sign is given in terms of the flow velocity towards left) and increases toward the wall along the y-direction (width of the microchannel). Therefore, looking from the cilium to the upper wall along the width of the microchannel a red region is shown initially followed by the blue region before reaching the upper wall. The same concept can be applied to other regions in both Mode II and Mode III.

Quantitatively, along the vertical line AB at a location of 0.25 mm, both the velocity and vorticity profiles were measured and calculated, as shown in Fig. 4 right column. In Mode I, the absolute average *V*_*x*_ and *V*_*y*_ values were accounted to 1.51 ± 0.59 mm/s and 0.41 ± 0.16 mm/s, respectively, and a parabolic velocity profile along x-direction was evident. The magnitude of vorticity is accounted to be highest as 21.3 s^−1^ near the microchannel wall whereas accounted to be negligible at the center of the microchannel. However, the absolute average *V*_*x*_ and *V*_*y*_ values were quantified to be 0.87 ± 0.76 mm/s and 0.12 ± 0.11 mm/s in Mode II whereas they are 1.46 ± 0.70 mm/s and 0.43 ± 0.37 mm/s in Mode III. Relative large values in *V*_*x*_ and *V*_*y*_ are evidenced for Mode III in comparison to Mode II which indicates certain flow fluctuations enhancing the mixing intensity, and thus, it may lead to superior sperm activation. In addition to that, along the line AB, the maximum absolute vorticity for Mode II and Mode III were accounted as 52.66 s^−1^ and 42.30 s^−1^, respectively. Additionally, the strength of vortex in the vicinity of artificial cilia was quantified based on the circulation along the designated closed curves (left column of Fig. 4b,c) for the Mode II and Mode III. Specifically, a circular zone of 30 µm was created in the vicinity of cilia, and the vorticity was integrated through the circular zone to obtain the circulation value. For Mode II and Mode III, the circulation values were quantified as 0.044 mm^2^/sec and 0.083 mm^2^/sec respectively at 0.75 T (T refers to the time required for each artificial cilia rotation). The circulation value for Mode III is approximately two times more than Mode II in terms of magnitude illustrating the significant local rotation in the flow field. As quantified, the magnitude of both the vorticity and circulation value found to be maximum for Mode III highlights the importance of additive stirring towards micromixing at an environment of low Re number.

Still, these flow differences do not specifically provide a convincing concept illustrating the reason why there is superior sperm activation in Mode III than that in Mode II since the flow data are quite comparable (no huge difference) between both modes. A follow-up discussion therefore was initialized in the following with an emphasis on the change of the time-dependent vorticity distribution exclusively found only in Mode III.

### Time lapse induced flow field comparison through artificial cilia beating cycle

**Figure 5:**
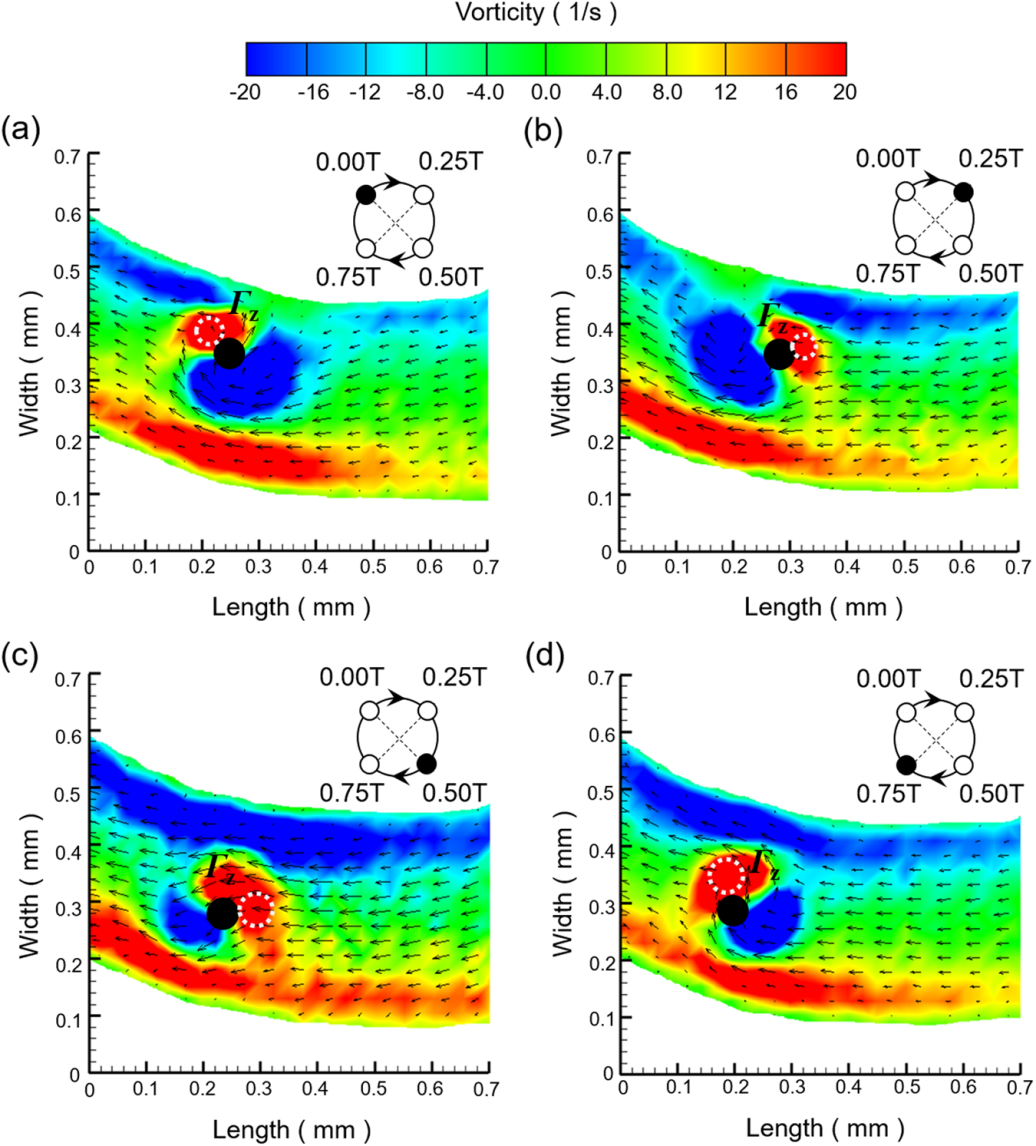
(**a**–**d**) Time-ensembled vorticity contour superimposed with mean velocity vectors at four different time points in a rotational cycle of artificial cilia for Mode III. The black circle represents the physical presence of an artificial cilium. The white dashed circle refers to the area calculated for circulation calculation.

To provide quantitative description of the induced flow vorticity change over time due to the actuation of artificial cilia, time-ensembled vorticity contours superimposed with the mean velocity vectors at four-time points (0 T, 0.25 T, 0.5 T and 0.75 T) in a typically rotational cycle of artificial cilia for Mode III are plotted in Fig. 5. As illustrated, two clearly distinguishable vorticity regions of peak intensity with the opposite signs are evidenced in the vicinity of artificial cilia. Specifically, in this mode with the progress of time and the change in cilia position, these vorticity distribution patterns were progressively altered. Upon quantification, the circulation values at the four respective time points were quantified as 0.11, 0.09, 0.08, and 0.15 mm^2^/sec, respectively. This change in vorticity pattern can be observed only in Mode III as in Mode II the vorticity distribution does not change over time since all artificial cilia are in static positions. It is believed that such unique flow feature found in Mode III contributes significantly to the superior sperm activation through the propagation of the vorticity pattern over time. This time change in vorticity pattern facilitates the local-axis rotation of sperm cells and the surrounding buffer flow which paves the roads for achieving high sperm activation rate. Such specific flow pattern manipulation is extremely beneficial in addition to the microchannel geometrical design as the activation rate can be increased further to over 74% as demonstrated in this study.

## Conclusion

In this work, an artificial cilia embedded serpentine microchannel was designed for the zebrafish sperm cell activation. The curvilinear design of the microfluidic device induces a rotational velocity field promoting global rotation whereas the artificial cilia motion instigates a local rotation along the center of the microchannel where artificial cilia are located. By harnessing both the global and local rotational flows, a reproducible mixing profile was achieved. It was found that with the proposed device an average 74.44% of the zebrafish sperm was activated within the first 15 s which is far more superior to its existing counterparts. The high efficiency of the proposed device demonstrated in this work sheds light into a promising future development in the field of germplasm physiology and activation of cryopreserved sperm.

### Ethics statement

All the experiments were performed in accordance with the relevant guidelines and regulations, reviewed and approved by the *Institutional Animal Care* and *Use Committee* (IACUC) of National Cheng Kung University (Approval Number: 104103).
